# Emotion Regulation in Generalized Anxiety and Social Anxiety: Examining the Distinct and Shared Use of Emotion Regulation Strategies

**Published:** 2018-07

**Authors:** Imaneh Abasi, Behrouz Dolatshahi, Shirin Farazmand, Abbas Pourshahbaz, Shima Tamanaeefar

**Affiliations:** 1 Department of Clinical Psychology, Taleghani Educational Hospital, School of Medicine, Shahid Beheshti University of Medicine Sciences, Tehran, Iran.; 2 Department of Clinical Psychology, University of Social Welfare and Rehabilitation Sciences, Tehran, Iran.; 3 Department of Clinical Psychology, Roozbeh Psychiatry Hospital, Tehran, Iran.

**Keywords:** *Attention*, *Emotion Regulation*, *Generalized Anxiety Disorder*, *Rumination*, *Social Anxiety Disorder*

## Abstract

**Objective:** According to the transdiagnostic view, emotion regulation strategies are common among emotional disorders. Despite the vast majority of studies demonstrating the common role of emotion regulation strategies in emotional disorders including generalized anxiety disorder (GAD) and social anxiety disorder (SAD), distinct functions of these strategies are largely unknown. So, the aim of the present study was to assess the distinct and shared use of emotion regulation strategies in individuals with GAD and SAD symptoms.

**Method** **:** Participants were recruited from a community sample of Tehran using purposive sampling method. The sample (N = 346) consisted of 269 nonclinical individuals, 47 individuals with generalized anxiety symptoms, and 30 individuals with social anxiety symptoms. Informed consent was obtained from all the participants and they were asked to fill in a series of self -report questionnaires including GAD-IV, SIAS, DERS, EQ, ACS, and RRS. Data were analyzed by discriminant function analysis using SPSS-23.

**Results: **Findings revealed significant differences between the 3 groups in non-acceptance, focusing, shifting, brooding, and decentering (P<0.01). Results of discriminant function analysis showed 2 functions: the first accounted for 87.4% of the variance (Wilk`s Lambda = 0.81, df = 10, P<0.01, χ2 = 70.06), and the second for 12.6% of the variance (Wilk`s Lambda = 0.97, df = 4, P<0.05, χ2 = 9.43) of between group variability. Non-acceptance and focusing discriminated GAD and SAD the most.

**Conclusion: **The findings of the present study support both splitter and lumper approaches as there were some emotion regulation strategies common between GAD and SAD, including shifting, decentering, and brooding. However, some of them were distinct, such as focusing and non-acceptance. Nevertheless, there are still inconsistencies in research about the findings, and thus examining the pathways may illuminate these findings.

Historically diagnostic classification has witnessed two diverse viewpoints: “splitter approach”, which assumes that boundaries are needed between different types of disorders (1), and “lumper approach” that emphasizes common dimensions across psychopathologies (2, 3). Transdiagnostic approach has been developed to seek a shared process across highly comorbid disorders, especially emotional disorders, such as depression, generalized anxiety disorder, and social anxiety disorder, which is in line with lumper point of view (4, 5) and National Institute of Mental Health (NIMH) recent project, Research Domain Criteria (6). 

 As emotional problems are prominent in various clinical conditions, recently emotion regulation, as a transdiagnostic common factor, has been evaluated by many studies in different disorders (7-9). 

Emotion regulation is defined as an individual’s emotions and when and how he/she experiences and expresses them (10-12). Emotion regulation strategies are processes by which individuals modify their emotions; these strategies may be adaptive or maladaptive and are related to development and maintenance of a wide range of pathologies (7, 13-17). 

Modal model of emotion regulation posits that there are 5 distinct emotion regulation processes that occur at 5 points in time. These emotion regulation strategies are categorized into 2 groups: (1) antecedent focused strategies, including situation selection, situation modification, attentional deployment, cognitive change; and (2) response focused strategies, such as response modulation (11, 12). Building upon the process model, Mennin and Fresco have assumed that regulatory strategies can be distinguished from less elaborative regulatory components (attending, being aware) to more elaborative regulatory components (reframing/ reappraisal, distancing) based on the degree of regulatory efforts (3). 

The relationship between majority of emotional problems and maladaptive emotion regulation strategies is evident (18-21), however, their high associations with emotional disorders have been more scrutinized and reported (22, 23). 

Among emotional disorders, generalized anxiety disorder and social anxiety disorder highly co-occur (24, 25). Furthermore, they are highly prevalent, chronic and impose problems in almost all aspects of life and reduce quality of life (26-29). Thus, assessing the role of emotion regulation strategies, especially ones that have been suggested to relate to dysfunctions in SAD and GAD, is pivotal. With respect to the mentioned emotion regulatory strategies in emotion regulation model proposed by Mennin and Fresco, several studies have demonstrated the relationship among SAD and GAD and rumination (30-34), decentering (35-37), non-acceptance (38), and attentional control (39-41). 

According to the transdiagnostic and lumper approaches, these emotion regulation strategies are common among psychological disorders and a great number of studies have determined the contributing role of these strategies in various clinical disorders. Shared function of emotion regulation is not disputable, but the distinct role of emotion regulation strategies is ill-defined. Some recent movements have tried to clarify distinct roles of emotion regulation strategies in anxiety disorders including SAD and GAD (22, 38 and 42). Taking a middle ground between the 2 approaches (splitter and lumper) and in connection with previous researches, the purpose of the present study was to seek the distinct and shared use of emotion regulation strategies in individuals with SAD and GAD symptoms. Thus, the study questions are that which emotion regulation strategies are common and which are distinct between individuals with GAD and SAD.

## Materials and Methods

Participants were recruited from a community sample of Tehran, Iran, using purposive sampling method. We can use any sample size for discriminant function analysis (DFA), but the sample size of the smallest group should exceed the number of predictive variables, for example, if we have 5 predictive variables, the sample size of the smallest group should exceed 5 (43). The sample (N = 346) consisted of 269 nonclinical individuals, 47 individuals with generalized anxiety symptoms, and 30 with social anxiety symptoms. Inclusion criteria were age ≥18 and participating voluntarily in the study. Exclusion criteria for the nonclinical population were scores equal or above 10 in GAD-IV, and 34 in SIAS. Cut off scores for questionnaires were, ≥10 (44), and ≥34 (45) for diagnosing GAD, and social anxiety, respectively.

***Procedure***

Participants were recruited by advertisements and in places where people gathered such as coffee shops and parks. The participants were informed about the purpose of the research and were asked to participate in the study that took place in the clinics in a quiet room. Participation was voluntary. After completing the informed consent, participants were asked to fill in a series of self -report questionnaires. After gathering the questionnaires, data of 500 participants were entered in to SPSS-23, and after data cleaning (removing missing and outliers), data of 480 participants remained. All individuals with exclusion criteria were removed from the analysis and at last 346 individuals remained. 

***Data Analysis***

Data were analyzed using chi- square to assess the differences between groups in gender and education level, and descriptive analysis was used to obtain descriptive statistics. ANOVA was utilized to assess the differences between groups in age. Finally, discriminant function analysis was run to evaluate whether there were differences between groups in emotion regulation strategies and which ones most discriminated GAD and SAD.

***Measures***

Social Interaction Anxiety Scale (SIAS): SIAS is a 20-item self-report questionnaire, which is rated on a 5- point Likert scale, ranging from 0 (not at all characteristic or true of me) to 4 (extremely characteristic or true of me). Internal consistency of SIAS in social phobia sample, community sample, and undergraduate sample has been reported as 0.86, 0.95, and 0.85, respectively (45). Iranian psychometric properties has been demonstrated to have acceptable internal consistency (α = 0.90) and test-retest reliability (r = 0.79) (46). Internal consistency of SIAS in the present study was α = 0.88.

Generalized Anxiety Disorder (GAD-7(44): GAD-7 is a self-report 7- item scale that assesses the severity of generalized anxiety disorder and is rated on a 0 (not at all) to 3 (nearly every day) rating scale. Internal consistency and test-retest reliability have been reported to be α = 0.92 and r = 0.83, respectively. Cut off point of 10 has been identified with optimized sensitivity (89%) and specificity (82%) (44). Cronbach alpha of GAD-7 in the present research was α = 0.89.

Attentional Control Scale (ACS): ACS is a 20- item self-report questionnaire, which is rated on a 4- point Likert scale (1 = almost never to 4 = always) and assesses attentional control and attentional shifting; its internal consistency has reported to be α = 0.88 (47). Test-retest reliability for ACS varies from 0.45 to 0.73 and internal consistency for the total score is 0.61 (48). Coefficient alpha for the focusing, shifting, and the total score of ACS was α = 0.78, α = 0.66, and α = 0.77, respectively (49). Internal consistency of focusing and shifting subscales in the present study was 0.75 and 0.67, respectively. 

The Ruminative Response Scale (RRS): RRS is a 22- item self-report questionnaire, which assesses the tendency to ruminate in response to depressed mood and is rated on a 4-point Likert-type scale, ranging from 0 to 3. Internal consistency (α = 0.89) and 5- month test- retest reliability have been reported to be acceptable (50, 51). Internal consistency of the Persian version of this scale is reported to be 0.81 (52). Internal consistency of Brooding subscale in the present study was α = 0.70.

Difficulties in Emotion Regulation Scale (DERS): DERS is a 36-item self-report scale and assesses individual’s typical tendencies for emotion regulation across several facets: (1) non-acceptance of emotional responses, (2) difficulties engaging in goal directed behavior, (3) impulse control difficulties, (4) lack of emotional awareness, (5) limited access to emotion regulation strategies, and (6) lack of emotional clarity. DERS demonstrates high internal consistency (α = higher than 0.80 for each subscale), good test-retest reliability (r = 0.88), and adequate construct and predictive validity (53). Psychometric properties of the Persian version has reported high internal consistency (α = 0.86), with acceptable correlation with Zuckerman-Kuhlman Personality Questionnaire (54). Non-acceptance subscale was used in the present research. Internal consistency of non-acceptance subscale in the present study was α = 0.84.

Experiences Questionnaire (EQ): EQ is an 11- item self-report questionnaire, which is rated based on a 7-point Likert scale, ranging from 1(never) to 7 (all the time) and assesses decentering. Internal consistency and test-retest reliability of this questionnaire have been demonstrated to be α = 0.89 and r = 0.87 (55). Internal consistency of decentering in Iranian population was acceptable, α = 0.82 (56). Internal consistency of EQ in the present study was α = 0.93.

## Results

***Descriptive Statistics***

No significant differences were found between groups in age (F (2, 343) =1.65, P<0. 19), gender (P<0.13), and educational level (P<0.73). Mean age for GAD, SAD, and control groups was 35.87 (SD = 11.4), 39 (SD = 10.38), and 35.25 (SD = 10.63), respectively. Descriptive statistics of the demographic variables are presented in Table 1.

***Discriminant Function Analysis***

Before performing discriminant function analysis, assumptions underlying DFA were examined and all assumptions, including absence of outliers, absence of missing data, linearity, normality, and absence of multicollinearity, were met. Discriminant function analysis using enter method revealed significant differences between groups in independent variables, including focusing, shifting, decentering, brooding, and non-acceptance, and these variables had significantly contributed to function (P<0.01). The summary results of equality of group means, along with the descriptive statistics of the study variables, are presented in Table 2. DFA revealed 2 functions and both were significant (F1 = P<0.01, F2 = P<0.05). The summary results of discriminant function analysis are demonstrated in Table 3. The 2 functions accounted for 87.4% and 12.6% of the between group variability, respectively. Standardized discriminant function coefficients showed the relative importance of each predictor in predicting each group from each function. Standardized discriminant function coefficients suggested that best predictors for distinguishing between GAD and SAD groups were focusing and non-acceptance, respectively. Overall, the discriminating power is acceptable and 59.2% of the original group cases were classified correctly.

## Discussion

The aim of the present study was to examine the common and specific patterns in levels of emotion regulation strategies including focusing and shifting as subscales of attentional control, brooding, decentering, and non-accepting in GAD, SAD, and control groups. Wilk`s lambda showed significant differences among the 3 groups in emotion regulation strategies. Focusing and non-acceptance best discriminated the SAD and GAD groups. 

Attentional control has been indicated in etiology and perpetuation of anxiety disorders, especially SAD and GAD that is in line with the results of the present research demonstrating its transdiagnostic role in psychopathology (40, 57 and 58). In fact, comorbidity of GAD and SAD may suggest the existence of shared dysfunction in the brain supporting the attentional control as a common emotion regulation strategy (59). Other emotion regulation strategies were significantly higher in SAD and GAD than the control group, which support the transdiagnostic nature of these variables and present findings that are consistent with those of previous studies, revealing the relationship among decentering, brooding and non-acceptance with GAD and SAD (31, 34, 35, 37, 38, 60, and 61). 

Among the emotion regulation strategies examined, focusing and non-acceptance discriminated individuals with GAD symptoms and individuals with SAD symptoms. In line with the present study, it has been demonstrated that patients with generalized anxiety disorder, in contrast to patients with generalized social anxiety disorder, show greater impairments in the recruitment of regions implicated in top-down attentional control (59). Despite the vast majority of studies ranging from very primitive to very complicated that explored and demonstrated the role of attentional bias as a very closed concept to attentional control in emotional disorders (62-64), findings of the present study support the disorder-specific nature of attentional control.

The distinct role of acceptance in anxiety disorders has not been evaluated enough and there are not adequate studies to refer to. One study found that non-acceptance of emotion best predicts the comorbidity of GAD and SAD (38), which is somewhat contradictory to findings of the present research. This disagreement may be due to different sample groups and different methods of choosing the samples. Furthermore, individuals with SAD pay less attention to emotions than individuals with GAD (42) and lack of interactions with others hold them back from understanding their emotions, so they are less acceptance of their emotions (38). 

Non-acceptance and attentional control as 2 major shared and yet distinct factors in anxiety disorders have captured the attention of the researchers and clinical psychologists who try to shed light on the mechanism and mediating functions of these variables in emotional disorders. Two well-known and qualified treatments that are widely used to help individuals suffering from anxiety and mood disorders and other psychological disorders are mindfulness and acceptance- based interventions, which largely emphasize improving acceptance and attention (65-68). Thus, given the importance of acceptance and attention in psychopathology and treatment of SAD and GAD, especially acceptance in SAD and attention in GAD, paying more attention to them and taking steps to use them in various studies on GAD and SAD are necessary and can illuminate vague points in recovering, improve quality of life, and increase productivity. 

Overall, findings of the present study are consistent with those of the previous studies, indicating that transdiagnostic components are not cultural phenomena and are not different across various cultures. There were not any significant differences between the study variables in nonclinical and clinical groups, which strengthens the transdiagnostic nature of the study variables. 

**Table 1:** Descriptive Statistics of the Demographic Variables of Control, GAD and SAD Groups

		**Controls**	**GAD**	**SAD**
**Gender**				
	Male	125 (46.5%)	21 (44.7%)	16 (53.3%)
	Female	144 (53.5%)	26 (55.3%)	14 (46.7%)
**Marital status**				
	Single	82 (30.5%)	15 (31.9%)	10 (33.3%)
	Married	183 (68%)	31 (66%)	17 (56.7%)
	Divorced	3 (1.1%)		2 (6.7%)
	Others	1 (0.4%)	1 (2.1%)	1 (3.3%)
**Education level**				
	Less than a high school diploma	30 (11.2%)	8 (17%)	6 (20%)
	High school diploma	115 (42.4%)	20 (42.6%)	13 (43.3%)
	Bachelor	81 (30.1%)	15 (31.9%)	6 (20%)
	Master	37 (13.8%)	3 (6.4%)	3 (10%)
	Doctoral	6 (2.2%)	1 (2.1%)	2 (6.7%)

**Table 2:** Descriptive Statistics and Equality of Group Means of Emotion Regulation Strategies in Clinical and Control Groups

** Mean (SD)**				**Equality of Group Means**		
	**GAD**	**SAD**	**Control**	**Wilks Lambda**	**F**_(2, 343)_	**sig**
**Focusing**	24.08 (4.3)	23.53 (4.3)	27.06 (3.69)	0.89	20.95	0.01
**Shifting**	18.78 (3.69)	17.63 (3.74)	19.6 (3.81)	0.97	4.19	0.04
**Decentering**	34.72 (7.9)	34.46 (5.59)	38.07 (6.39)	0.95	8.33	0.01
**Brooding**	11.46 (2.12)	10.63 (2.52)	9.72 (2.64)	0.94	10.08	0.01
**Non-acceptance**	13.87 (5.26)	16.53 (4.57)	11.86 (4.67)	0.91	15.2	0.01

**Table 3:** Summary Results of Discriminant Function Analysis of Emotion Regulation Strategies in Clinical and Control Groups

	**Function 1**	**Function 2**
**Structure matrix**		
**Focusing**	0.79	0.05
**Shifting**	0.33	-0.31
**Decentering**	0.49	0.10
**Brooding**	-0.49	-0.61
**Non-acceptance**	-0.63	0.57
**Eigenvalue (variance %)**	0.19 (87.4%)	0.02 (12.6%)
**Canonical correlation**	0.40	0.16
**Wilks lambda**	0.81	0.97
**Chi-square**	70.06	9.43
**df**	10	4
**sig**	0.01	0.05
**Group centroids**		
**GAD**	-0.72	-0.31
**SAD**	-0.95	0.4
**Nonclinical**	0.23	0.01

## Limitation

A number of limitations in the present study suggest that findings should be interpreted cautiously. First, the present sample was selected from community individuals who had higher functioning and less impairment in different life domains and had less severe clinical symptoms. Therefore, future studies on clinical patients may yield more realistic and reliable findings. Second, exclusively relying on self-report measures may subject the findings to considerable bias, especially about emotion regulation related concepts, as sometimes it is difficult to grasp their meaning, so future studies may benefit from conducting multi-assessment procedures including physiological and neurobiological instruments to assess emotion regulation strategies. Third, this study was conducted on a restricted group of emotion regulation strategies. Thus, in future studies attention should be paid to different emotion regulation strategies between GAD and SAD, and also between other emotional disorders. Finally, due to the complicated contributory role of emotion regulation constructs in psychopathology, studying them as mediators and moderators may illuminate more straightforward and cost-effective conceptualization and treatment.

## Conclusion

Focusing and non-acceptance were highlighted in distinguishing SAD and GAD symptoms in the present study. Moreover, as non-acceptance is much higher in individuals with SAD symptoms than in those with GAD symptoms, other related psychological factors responsible for this connection should be taken into consideration in future studies.
